# Noninvasive Genetic Assessment Is an Effective Wildlife Research Tool When Compared with Other Approaches

**DOI:** 10.3390/genes12111672

**Published:** 2021-10-23

**Authors:** Miriam A. Zemanova

**Affiliations:** 1 Centre for Compassionate Conservation, School of Life Sciences, University of Technology Sydney, Ultimo, NSW 2007, Australia; miriam.andela.zemanova@gmail.com; 2 Animalfree Research, Postgasse 15, 3011 Bern, Switzerland; 3 Oxford Centre for Animal Ethics, 91 Iffley Road, Oxford OX4 1EG, UK

**Keywords:** animal welfare, diet analysis, DNA sampling, health monitoring, invasive research, population size estimation, species detection, wildlife genetics

## Abstract

Wildlife research has been indispensable for increasing our insight into ecosystem functioning as well as for designing effective conservation measures under the currently high rates of biodiversity loss. Genetic and genomic analyses might be able to yield the same information on, e.g., population size, health, or diet composition as other wildlife research methods, and even provide additional data that would not be possible to obtain by alternative means. Moreover, if DNA is collected non-invasively, this technique has only minimal or no impact on animal welfare. Nevertheless, the implementation rate of noninvasive genetic assessment in wildlife studies has been rather low. This might be caused by the perceived inefficiency of DNA material obtained non-invasively in comparison with DNA obtained from blood or tissues, or poorer performance in comparison with other approaches used in wildlife research. Therefore, the aim of this review was to evaluate the performance of noninvasive genetic assessment in comparison with other methods across different types of wildlife studies. Through a search of three scientific databases, 113 relevant studies were identified, published between the years 1997 and 2020. Overall, most of the studies (94%) reported equivalent or superior performance of noninvasive genetic assessment when compared with either invasive genetic sampling or another research method. It might be also cheaper and more time-efficient than other techniques. In conclusion, noninvasive genetic assessment is a highly effective research approach, whose efficacy and performance are likely to improve even further in the future with the development of optimized protocols.

## 1. Introduction

The global change and decline of biodiversity require effective species management based on continuous monitoring of trends within wildlife populations [1]. Monitoring of animal populations can be conducted in numerous ways, for instance, through capture-mark-recapture [2], camera traps or aerial surveys [3,4], radio or GPS tagging [5,6], counting of traces such as faeces and burrows [7,8], or through genetic assessment [9].

Genetic monitoring in particular can be a powerful research tool, as it is capable of providing the same information as other methods, for instance, population size estimates [10,11], species detection [12,13], individual identification [14,15], or diet composition [16,17,18]. Moreover, DNA analyses can deliver multitude of data that might be difficult or impossible to obtain with other methods, e.g., on relatedness among individual animals [19,20], population structure [21,22,23], origin of invasive species [24,25,26,27], hybridization [28,29,30,31], past and present population sizes [7,32,33], or gene flow [26,27,31,34,35,36,37,38,39].

Traditionally, DNA samples have been obtained from blood or tissues [40]. The advantage of these samples is that they contain high-quality DNA in large quantities, the disadvantage is the invasiveness of these methods, with potentially negative implications for animal welfare [40]. Fortunately, DNA samples can be also collected in a way that requires no or only minimal physical interaction with the animal. Noninvasive genetic sampling *sensu lato* is defined as “obtaining DNA without affecting the physical integrity of the animal through puncturing the skin or other entry into the body” [41]. This approach was first used approximately 30 years ago, to obtain DNA from hair samples of chimpanzees (*Pan troglodytes*) [42] and faecal samples of brown bears (*Ursus arctos*) [43]. Although faeces and hair remain commonly used sources of non-invasively obtained DNA, it is now possible to obtain genetic sequences also from feathers, saliva, urine, slime, or eggshells [44]. Furthermore, recent advances in sequencing technologies allow for detecting the presence of a target species or even for describing whole communities by metabarcoding of the so-called environmental DNA (eDNA) samples from water [45,46] or soil [47]. In this approach, a sample is amplified using primers for a standard barcode region, such as the mitochondrial COI, and sequenced on a high-throughput sequencing platform [48].

Nevertheless, the proportion of genetic studies using noninvasive sampling is still relatively low [9]. Furthermore, it remains unclear in which contexts is noninvasive genetic assessment better suited to provide the relevant data than alternative research methods that are not based on DNA analysis. Broader implementation of noninvasive genetic sampling instead of invasive and lethal sampling or other approaches might be dependent on the perception of its effectiveness, suitability, costs and time effort. However, a comprehensive assessment of the performance or efficacy of noninvasive genetic assessment in comparison with other methods has never been done before. Therefore, the objective of this paper was to review and compare the performance of noninvasive genetic assessment and other research methods across a wide range of different types of studies, animal species, and sources of non-invasively sampled genetic material.

## 2. Materials and Methods

The research was conducted following the Preferred Reporting Items for Systematic Reviews and Meta-Analyses (PRISMA) guidelines [49]. Three of the largest databases that should cover most of the topic-specific studies were used for identifying the relevant scientific literature [50]: Web of Science (1900–2020), SCOPUS (1970–2020), and Agricultural and Environmental Science Collection (1970–2020). A preliminary search was used to identify keywords that were likely to capture most relevant studies, without being too general. The final search strings modified for each database to reflect the different abbreviations used are listed in Table 1. The search was conducted on the 15 February 2021.

**Figure 1:**
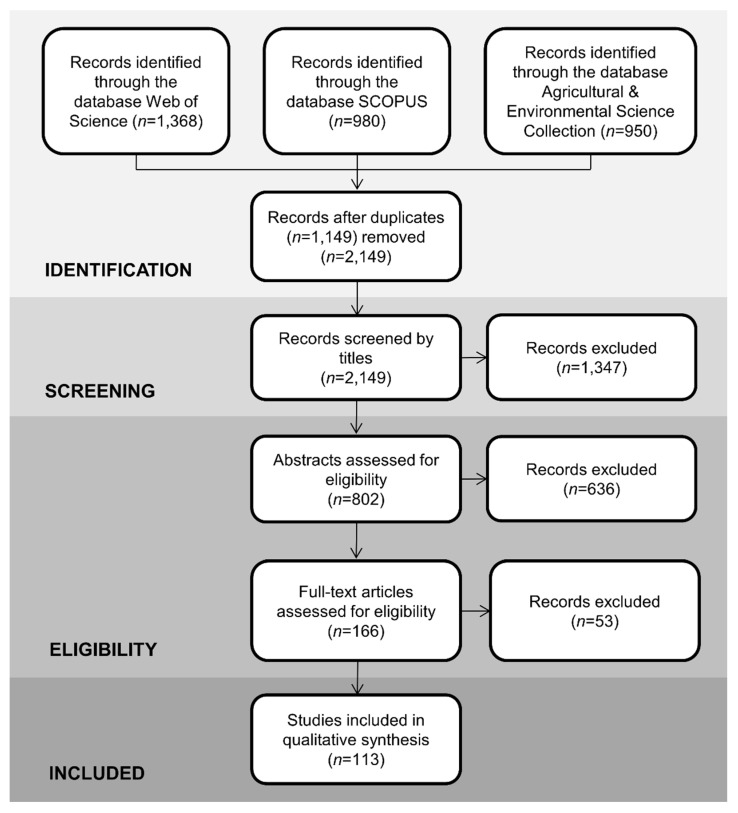
PRISMA literature search flow diagram. The number of studies (*n*) that were identified, screened, retained, or discarded are shown at each stage of the review process.

After exclusion of duplicate records (Figure 1), a three-stage assessment was performed of whether to include the study in the review: (1) selection by title (2) selection by abstract, and (3) selection by the content of the full text if the abstract did not provide enough detail to select study in the previous stage.

The inclusion criteria were (a) studies that contained quantitative data on comparison of noninvasive genetic assessment *sensu lato* with another research method, drawing conclusions on their performance, (b) peer-reviewed studies, and (c) studies published in English. The exclusion criteria were (A) studies in other fields than wildlife research, (B) studies comparing two noninvasive genetics methods, (C) studies without comparison, (D) reviews, (E) commentaries, editorials, conference abstracts, or (F) book chapters.

To check the suitability of the exclusion criteria, a random subset of 5% of all studies generated by the search was sent to an external reviewer. The reviewer was instructed to exclude or include the studies based on the criteria listed above using the title and abstract, and if necessary reading the full text. The percentage agreement between the author and the reviewer was 98.2%. Thus, the exclusion criteria were considered repeatable and rigorous.

From each study identified as relevant for the review, the following data were extracted: (1) year when the study was conducted, (2) country of field or lab work, (3) target animal species, (4) type of the study (e.g., species identification, population size estimation, etc.), (5) source of non-invasively obtained DNA samples (e.g., faeces, feather, hair, etc.), (6) research method the noninvasive genetic assessment was compared to (e.g., invasive genetic sampling, camera traps, field survey, etc.), (7) performance of the noninvasive genetic assessment in comparison with the other research method (e.g., in terms of ability to obtain genotypes, accuracy in population size estimates, number of detected species, etc.), and if available, also comparisons of (8) costs, and (9) time effort of either method.

## 3. Results

**Figure 2:**
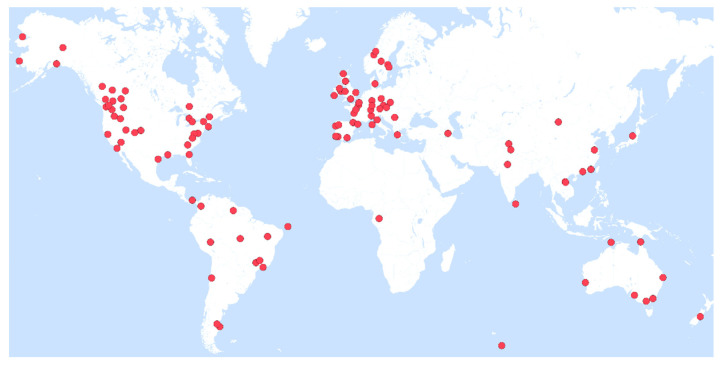
Locations of the field or laboratory work of the 113 studies identified in this study.

**Figure 3:**
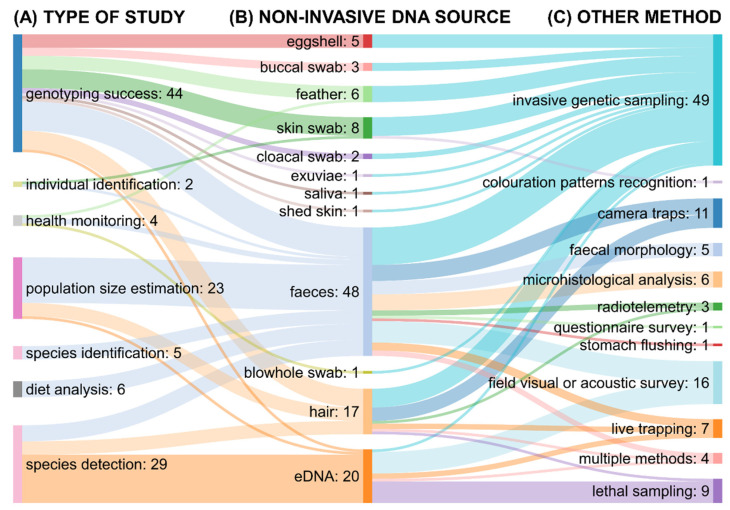
Sankey diagram with the number of studies grouped according to the type of study (**A**), source of a non-invasively obtained DNA sample (**B**), and method compared to the non-invasive genetic assessment (**C**). The thickness of the lines linking categories is proportional to the number of studies and the colour corresponds to the target category going from left to right.

Using the three databases, 2149 unique records were retrieved (Figure 1). After conducting the assessment stages for each of the records based on the exclusion criteria, the present review consisted of 113 relevant studies (Table S1; Figure 1, Figure 2 and Figure 3).

### 3.1. Geographical and Temporal Patterns

**Figure 4:**
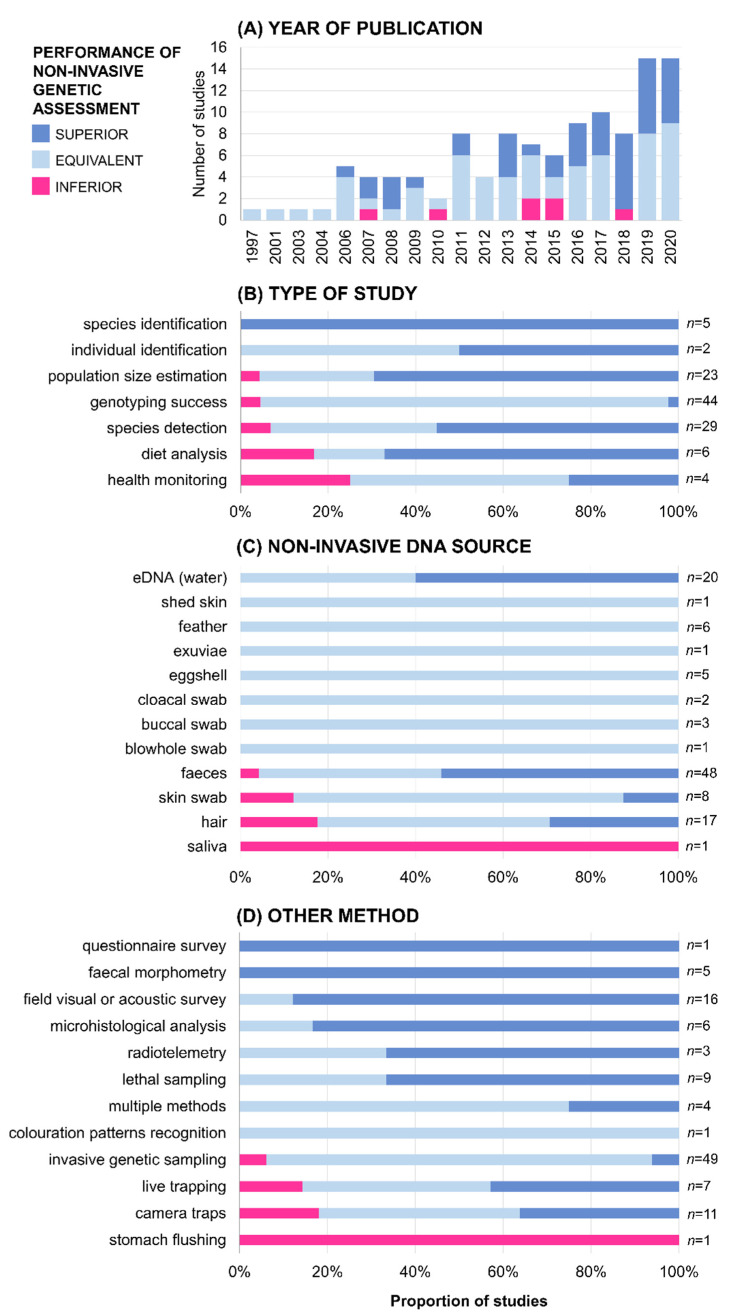
Performance of noninvasive genetic assessment across the 113 studies. (**A**) The number of studies included in the review published in 1997–2020. (**B**) The proportion of studies sorted by their type. (**C**) Sorted by the source of non-invasively obtained DNA sample used. (**D**) Sorted by the method that noninvasive genetic assessment was compared to.

Most of the studies were conducted in North America (USA: *n* = 28; Canada: *n* = 10; Table S1; Figure 2). Other regions with several studies included Australia (*n* = 9), Brazil (*n* = 6), and European countries (e.g., France: *n* = 6; UK: *n* = 6; Portugal: *n* = 4). Since the focus was on academic literature written in English, this geographical pattern mirrors the general pattern observed in field-based ecological studies [51]. Studies spanned the years 1997–2020 (Table S1). Across this period, the number of studies that met the inclusion criteria increased steadily over time with a peak in 2019–2020 with 15 studies (Figure 4).

### 3.2. General Characteristics of the Included Studies

The most studied animals were carnivores (*n* = 34), birds (*n* = 19), and fish (*n* = 12; Table S1). Seven general types of studies were identified (Figure 3). Most of the studies included in this review focused on assessing the genotyping success of non-invasively versus invasively obtained DNA samples (*n* = 44). The second and third most common types of studies were species detection (*n* = 29) and population size estimation (*n* = 23). As the source of non-invasively obtained DNA were used mostly faeces (*n* = 48), eDNA (*n* = 20), and hair (*n* = 17). Concerning the method, the noninvasive genetic assessment was compared to, invasive genetic sampling (*n* = 49), field visual or acoustic survey (*n* = 16) and camera traps (*n* = 11) were represented most frequently.

### 3.3. Performance of Noninvasive Genetic Assessment

Out of the 113 studies, 62 reported equivalent, 44 superior, and only 7 inferior performances of the noninvasive genetic assessment in comparison with another research method (Table S1; Figure 4).

#### 3.3.1. Comparison of Genotyping Success

In total, 39% of the studies included in this review focused on comparing the amplification and/or genotyping success of samples obtained non-invasively versus through invasive genetic sampling of blood or tissues (Table S1; Figure 3 and Figure 4). In this subset of studies, the sources of non-invasively obtained DNA were faeces (*n* = 11), skin swabs (*n* = 7), hair (*n* = 7), eggshells (*n* = 5), feathers (*n* = 5), buccal swabs (*n* = 3), cloacal swabs (*n* = 2), exuviae (*n* = 1), saliva (*n* = 1), eDNA (*n* = 1), and shed skin (*n* = 1; Figure 3). Out of the 44 studies in this category, 41 reported that the authors were able to obtain genotypes from both non-invasively and invasively sampled genetic material (Table S1; Figure 4). The study by Karlsson et al. [52] reported better efficacy of noninvasive genetic sampling in genotyping. The authors assessed four methods of DNA sampling in freshwater mussels *Margaritifera margaritifera*: haemolymph extraction, foot scraping, mantle biopsy, and skin swabbing. The genotyping success was lowest for the haemolymph extraction and mantle biopsy, i.e., when using invasive methods. Two studies reported inferior genotyping success of non-invasively obtained DNA samples (Figure 4). Duenas et al. [53] evaluated the use of saliva samples from brushtail possum (*Trichosurus vulpecula*) collected through wax tags. The comparison of amplification success with tissue samples showed poor performance of the non-invasively obtained DNA. However, the authors admitted that the saliva collection devices may not have been properly designed and the use of buccal swabs would have been more appropriate for DNA collection. The study by Ringler [54] tested skin swabbing in dendrobatid frog *Allobates femoralis*. Comparing the amplification success of test swabs versus tissue samples obtained through toe clipping, the author reported insufficient genotyping success of the noninvasive samples. The author described that this might have been the consequence of the relatively dry skin of the frog species and the presence of alkaloids in the skin [54].

#### 3.3.2. Species Detection

The second most common type of study was species detection (26%; Table S1; Figure 3 and Figure 4). Most studies in this subset used eDNA as the source of genetic material (*n* = 18), followed by faeces (*n* = 6) and hair (*n* = 5). Out of the 29 studies, 11 reported being able to detect equal number of species using either noninvasive genetic assessment or camera traps (*n* = 3), field visual or acoustic survey (*n* = 2), lethal sampling (*n* = 2), live trapping (*n* = 1), or a combination of multiple methods (*n* = 3). Sixteen studies stated that noninvasive genetic assessment was able to detect more species than field visual or acoustic survey (*n* = 7), lethal sampling (*n* = 5), live trapping (*n* = 2), invasive genetic sampling (*n* = 1), and camera traps (*n* = 1). Two studies reported lower detection rates of noninvasive genetic assessment. Fisher and Bradbury [55] compared the detection rate of hair traps versus camera traps for marten (*Martes americana*), fisher (*Pekania pennanti*), and wolverine (*Gulo gulo*). In this study, the cameras performed better than hair trapping and the authors, therefore, recommended using multiple independent survey methods. Monterroso et al. [56] also assessed the efficiency of hair traps and camera traps and reported that camera traps were a more efficient method for detecting mesocarnivores. The authors suggested that the poor performance of genetic assessment might have been caused by the low number of sampling occasions [56].

#### 3.3.3. Population Size Estimation

In total, 23 studies focused on population size estimation (Table S1; Figure 3 and Figure 4). The authors of six studies reported that they were able to obtain equivalent population size estimates with genetic assessment using faeces as the source of DNA material as with camera traps (*n* = 2), live trapping (*n* = 2), or radiotelemetry (*n* = 1), and when using eDNA versus lethal sampling (*n* = 1). The use of noninvasive genetic assessment provided more accurate population size estimates in 16 studies. Within these, DNA analysis obtained from faecal samples was compared with field visual or acoustic surveys (*n* = 7), camera traps (*n* = 2), invasive genetic sampling (*n* = 1), live trapping (*n* = 1), and a questionnaire survey (*n* = 1), DNA analysis obtained from hair samples with camera traps (*n* = 1), lethal sampling (*n* = 1), radiotelemetry (*n* = 1), and a combination of multiple methods (*n* = 1). Only one study on population size estimation showed an inferior performance of noninvasive genetic sampling in comparison with live trapping [57]. The authors investigated the population of spotted-tail quoll (*Dasyurus maculatus*) and deployed baited hair-sampling devices in which the capture rate of hair samples was rather low. As suggested by the authors, improvements to the hair-sampling method would have allowed for better sampling efficacy [57].

#### 3.3.4. Diet Analysis

All six studies focusing on the analysis of dietary composition used metabarcoding of faeces (Table S1; Figure 3 and Figure 4). This method did not work well in the study by Deagle et al. [16], in which stomach flushing was more precise in identifying the diet of macaroni penguins (*Eudyptes chrysolophus*). However, the authors mentioned problems with laboratory protocols and still recommended using noninvasive genetic assessment. In other studies on dietary composition, noninvasive genetic assessment performed equally well (*n* = 1) or even better (*n* = 4) when compared with microhistological analysis.

#### 3.3.5. Species Identification

Five studies compared the performance of methods used for species identification (Table S1; Figure 3 and Figure 4). Noninvasive genetics approach using faecal samples allowed for a more accurate species identification than faecal morphometry in all five studies.

#### 3.3.6. Health Monitoring

There were four studies included in this review that compared the performance of the noninvasive genetic approach and another method for health monitoring (Table S1; Figure 3 and Figure 4). Baek et al. [58] assessed the detection of Avipoxvirus in different samples taken from hummingbirds. They found that feathers are equally reliable for detecting the virus as tissue samples. Equivalent performance was reported also in the study by Wu et al. [59], in which the authors compared the detection of bacteria *Brucella* spp. in bottlenose dolphins (*Tursiops truncates*) using blowhole swab and tissue samples. Bertram et al. [60] were able to detect more positive samples containing a bird parasite through PCR of faecal samples than through microhistological analysis. In contrast, Martinsen et al. [61] stated that for detecting malaria parasites in birds, faecal samples may not be suitable and recommended the use of blood.

#### 3.3.7. Individual Identification

Two studies focused on individual identification and neither reported inferior performance of the noninvasive genetic assessment approach in identifying specific individuals (Table S1; Figure 3 and Figure 4). DeMay et al. [15] described that faecal DNA sampling is better suited for longer-term monitoring of individual pygmy rabbits (*Brachylagus idahoensis*) than radiotelemetry. Monteiro et al. [14] were able to reliably identify individual pipefish (*Nerophis lumbriciformis*) both through colouration patterns and genetic data obtained through skin swabs.

### 3.4. Costs and Time Effort

**Figure 5:**
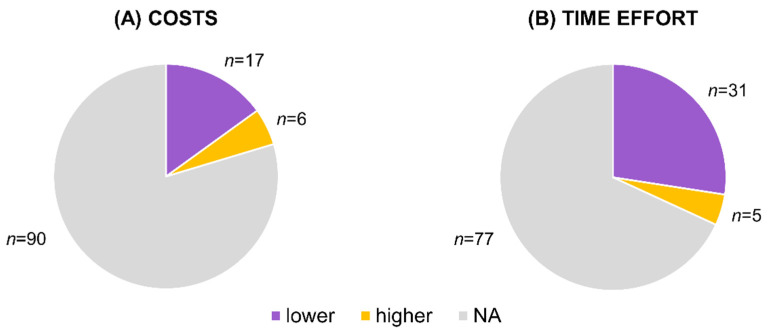
(**A**) The number of studies reporting lower or higher costs of noninvasive genetic assessment in comparison with another method and studies that did not make this comparison (NA = not assessed). (**B**) The number of studies reporting lower or higher time effort of noninvasive genetic assessment in comparison with to another method and studies that did not make this comparison (NA = not assessed).

Some of the studies also assessed the costs and time effort of the noninvasive genetic approach in comparison with another method (Table S1; Figure 5). Twenty-three out of the 113 studies evaluated the expenses, and 74% of these reported lower costs. Thirty-six out of the 113 studies compared the time effort, out of which 86% calculated that noninvasive genetic assessment requires less time than another approach. Specifically, several studies reported that noninvasive genetic assessment had lower costs and/or time requirements than radiotelemetry [15,62,63], microhistological analysis [64,65,66], lethal sampling [67,68], and live trapping [57,69,70,71,72]. The higher costs of invasive genetic sampling reported in some of the studies can be attributed to a more expensive equipment [37,73] and increased time required to sample the locations [74].

## 4. Discussion

### 4.1. Efficacy of Noninvasive Genetic Assessment

This review identified a wide range of wildlife research studies, ranging from assessment of genotyping success using a noninvasive sample versus invasively obtained samples, diet analysis, to population size estimation (Table S1; Figure 3 and Figure 4). Overall, 94% of the studies included in this review reported that noninvasive genetic assessment performs equally well or better than other approaches. Based on the reviewed studies, it can be particularly suitable for species or individual identification, population size estimation, species detection, and as an alternative to invasive DNA sampling (Figure 4). The 6% of the studies reporting inferior efficacy of noninvasive genetic sampling demonstrated that its performance strongly depends on the research aims and study design.

#### 4.1.1. Noninvasive vs. Invasive Genetic Assessment

The usually low quantity and quality of non-invasively obtained DNA samples require caution as these factors can cause a high error rate in genotypes [75]. Several studies included in this review therefore emphasized the importance of optimized laboratory protocols [16,75,76] as well as proper collection of samples [53,57,77,78].

Genomic techniques such as genome-wide sequencing have traditionally required a relatively high concentration of DNA. Even though this is still the case for some approaches, there have been recently developed new methods and protocols to accommodate low-quality or low-quantity DNA samples as well [44,79]. For instance, targeted sequencing using hybridization probes [80,81] and methylation-based enrichment techniques can be particularly effective [82]. The trend of laboratory protocols optimization has been visible in the reviewed studies. In the last five years, only one study out of 58 reported inferior performance of noninvasive genetic assessment in comparison with another method (Figure 4).

#### 4.1.2. Noninvasive Genetic Assessment vs. Other Research Approaches

The efficacy of the noninvasive genetic assessment might be superior to questionnaire surveys, faecal morphometry, field visual or acoustic surveys, microhistological analyses, radiotelemetry, or lethal sampling (Figure 4). Nevertheless, in particular circumstances, genetic assessment would have only limited use. For example, genetic analysis is not suitable to provide information on body weight [71], age [83], or behaviour [84]. Therefore, for some research goals, it would be most optimal to use a different approach, or a combination of noninvasive genetic sampling and another method, preferably also noninvasive [10,84,85,86].

### 4.2. Species Bias

Interestingly, carnivores seemed to be particularly popular among the included studies (Table S1). Similar findings were reported also in previous work assessing research on wildlife [9,87]. This might be caused by several factors. First, carnivores and large mammals in general are difficult to capture [88,89]. Secondly, their faces and hair might be relatively easy to find in comparison with those of smaller species [90,91]. In addition, lastly, some species are more sought-after study subjects than others [92,93] and attract most of the research and conservation funding [94]. Accordingly, the suitability of different methods might be understudied in certain animal groups and the same method may have different efficacy depending on the species studied [95].

### 4.3. Animal Welfare Considerations

One of the greatest challenges in wildlife research is to successfully monitor the target species or populations while causing minimal disturbance, stress or pain to the studied animal [40,96]. A huge benefit of noninvasive genetic assessment is the minimal or no impact on animal welfare, because depending on the method, it may not be necessary to even touch the animal [40]. Several studies included in this review explicitly mentioned no disturbance or harm to animals as an advantage over other methods [11,15,16,37,97,98,99,100]. However, tissue or blood sampling may not necessarily be an animal welfare issue, when samples are taken from an already deceased animal. An example of this is the use of museum samples or roadkill. Although none of the studies included in this review used this approach, it has been successfully implemented in, for instance, the assessment of genetic structure in the European hedgehog (*Erinaceus europaeus*) [101], kangaroo rat (*Dipodomys panamintinus*) [102], or Alabama red-bellied turtle (*Pseudemys alabamensis*) [103].

Furthermore, it is important to note that not all genetic sampling defined as noninvasive *sensu lato* can be considered harmless. Just capture of the animal—without affecting the physical integrity through a needle or another instrument—could be extremely stressful and might lead to capture myopathy [104,105]. Consequently, whenever possible, one should implement techniques that require no handling or disturbance of wildlife, such as the collection of faeces. In other cases, this would mean using a different approach, e.g., camera traps, which might be even more suitable than genetic assessment for reaching the specific research goals [106].

### 4.4. Cost and Time Effort Advantages

Noninvasive genetic assessment could be more cost- and time-effective than both invasive sampling and other survey methods (Figure 5). Nevertheless, the costs and time effort depend on the approach the noninvasive genetic assessment is being compared with, the type of study, and the research design (Table S1). For instance, field visual or acoustic survey used for population size estimation or species detection could be both less expensive [107,108] or more expensive [13,78,109,110,111] than noninvasive genetic sampling and analysis. Similarly, one study reported lower costs of camera traps for population size estimation [38], while two studies reported the opposite [112,113]. Another study showed that genotyping from non-invasively obtained DNA samples is more time-consuming and expensive than genotyping from blood or tissue samples [114], but four studies reported a contradictory calculation [37,73,74,91]. This variety in findings among the studies stresses the importance of optimal study design.

### 4.5. Study Limitations

The limits of the present study are related to restrictions in language and the search terms, such that potentially relevant studies might have been excluded. Furthermore, this review includes a wide range of studies with different study designs and target animal species and the results may not be always comparable. Similarly, due to the high heterogeneity among the relevant studies, it was not possible to quantitatively summarize the outcomes. The search should be nevertheless representative enough to provide a comprehensive overview of the currently available literature.

## 5. Conclusions

The evaluation of the efficacy of noninvasive genetic assessment is an important step toward a wider uptake of this methodology. This review constitutes the first effort to collate the peer-reviewed literature on the performance of noninvasive genetic assessment. The overwhelming majority of studies included in this review supported the notion that noninvasive genetic assessment is a very effective research tool, suitable for a large spectrum of wildlife studies. The recent technological advances in genetic sampling and sequencing methods provide new opportunities for fast, reliable, and cost-efficient wildlife research. Moreover, noninvasive genetic assessment is well-suited to address the increasing demand for effective and efficient research that has minimal impact on animal welfare.

## Figures and Tables

**Table 1:** Search strings used in each of the three databases. Search results were limited to research areas or topics specific to wildlife research.

Database	Search String	Limited to
Web of Science	AB = ((non-invasive OR noninvasive OR minimally invasive) AND (genetic* OR genomic OR DNA OR eDNA) AND (efficien* OR efficacy OR effect* OR perform* OR compar* OR validat* OR suitab*))	Research Areas: Zoology, Biodiversity Conservation, Evolutionary Biology, Environmental Sciences, Ecology, Genetics and Heredity
SCOPUS	ABS ((non-invasive OR noninvasive OR minimally invasive) AND (genetic* OR genomic OR DNA OR eDNA) AND (efficien* OR efficacy OR effect* OR perform* OR compar* OR validat* OR suitab*))	Research Areas: Agricultural and Biological Sciences, Environmental Science
Agricultural and Environmental Science Collection	ABSTRACT: ((non-invasive OR noninvasive OR minimally invasive) AND (genetic* OR genomic OR DNA OR eDNA) AND (efficien* OR efficacy OR effect* OR perform* OR compar* OR validat* OR suitab*))	Topics: Population Genetics, Invasiveness, Conservation, Wildlife, Genetic Diversity, Wildlife Conservation, Carnivores, Wildlife Management, Animal Populations, Biodiversity, Genetic Variation, Mammals, Endangered Species, Population

*: The asterisk serves as a wildcard operator that is used to broaden a search by finding words that start with the same letters.

## Data Availability

The data presented in this study are available in the Supplementary Material.

## Supplementary Materials

The following are available online at https://www.mdpi.com/article/10.3390/genes12111672/s1. Table S1: Relevant studies identified in the review, comparing the costs, time effort and performance of non-invasive genetic assessment (NGA).
