# Supplementary material for: Noninvasive Genetic Assessment Is an Effective Wildlife Research Tool When Compared with Other Approaches
Source: Genes (Basel). 2021 Oct 23;12(11):1672. doi: 10.3390/genes12111672 (PMC8625682; doi:10.3390/genes12111672)
Supplement: Supplementary file 1 [file genes-12-01672-s001.zip › genes-1397669-supplementary.pdf]

genes MDPI

Table S1. Relevant studies identified in the review, comparing the costs, time effort and performance of non-invasive genetic assessment (NGA).

Type of Study	Year	Country of Field or Laboratory Work	Target Animal	Non-Invasive DNA Source	Other Method	Costs	Time Effort	Efficacy of NGA	Reference
genotyping success	2013	Serbia	bird	feather	invasive genetic sampling	NA	lower	equivalent	[1]
genotyping success	2012	Ireland	bat	faeces	invasive genetic sampling	higher	higher	equivalent	[2]
genotyping success	2015	China	bird	feather	invasive genetic sampling	NA	NA	equivalent	[3]
genotyping success	2014	USA	carnivore	hair	invasive genetic sampling	NA	NA	equivalent	[4]
genotyping success	2015	New Zealand	marsupial	saliva	invasive genetic sampling	NA	NA	inferior	[5]
genotyping success	2009	Canada	bird	eggshell	invasive genetic sampling	NA	lower	equivalent	[6]
genotyping success	2020	USA	carnivore	hair	invasive genetic sampling	equivalent	NA	equivalent	[7]
genotyping success	2008	Brazil	cetacean	skin swab	invasive genetic sampling	NA	NA	equivalent	[8]
genotyping success	2003	Sri Lanka	elephant	faeces	invasive genetic sampling	NA	NA	equivalent	[9]
genotyping success	2017	Canada	reptile	cloacal swab	invasive genetic sampling	lower	lower	equivalent	[10]
genotyping success	2017	Colombia	bird	faeces	invasive genetic sampling	NA	lower	equivalent	[11]
genotyping success	2011	Germany	rodent	hair	invasive genetic sampling	NA	NA	equivalent	[12]
genotyping success	2012	Chile	amphibian	buccal swab	invasive genetic sampling	NA	NA	equivalent	[13]
genotyping success	2006	USA	bird	buccal swab	invasive genetic sampling	NA	lower	equivalent	[14]
genotyping success	2004	Sweden	carnivore	faeces	invasive genetic	NA	NA	equivalent	[15]

					sampling				
genotyping success	2017	USA	mollusc	skin swab	invasive genetic sampling	NA	NA	equivalent	[16]
genotyping success	2019	UK	mollusc	eDNA (water)	invasive genetic sampling	NA	NA	equivalent	[17]
genotyping success	2012	China	bird	faeces	invasive genetic sampling	NA	NA	equivalent	[18]
genotyping success	2013	Norway	mollusc	skin swab	invasive genetic sampling	NA	NA	superior	[19]
genotyping success	2020	India	carnivore	hair	invasive genetic sampling	NA	NA	equivalent	[20]
genotyping success	2001	France	primate	faeces	invasive genetic sampling	NA	NA	equivalent	[21]
genotyping success	2017	Brazil	bird	eggshell	invasive genetic sampling	NA	NA	equivalent	[22]
genotyping success	2011	Spain	bird	eggshell	invasive genetic sampling	NA	NA	equivalent	[23]
genotyping success	2019	USA	reptile	faeces	invasive genetic sampling	NA	NA	equivalent	[24]
genotyping success	2014	Switzerland	carnivore	hair	invasive genetic sampling	NA	NA	equivalent	[25]
genotyping success	2007	UK	ungulate	hair	invasive genetic sampling	lower	NA	equivalent	[26]
genotyping success	2016	Peru	bird	feather	invasive genetic sampling	NA	NA	equivalent	[27]
genotyping success	2020	Czech Republic	arthropod	exuviae	invasive genetic sampling	NA	NA	equivalent	[28]
genotyping success	1997	USA	bird	feather	invasive genetic sampling	NA	NA	equivalent	[29]
genotyping success	2020	Argentina	carnivore	hair	invasive genetic sampling	NA	NA	equivalent	[30]
genotyping success	2010	USA	primate	faeces	invasive genetic sampling	NA	NA	equivalent	[31]
genotyping success	2013	Austria	amphibian	skin swab	invasive genetic sampling	NA	NA	equivalent	[32]
genotyping success	2019	Ireland	ungulate	faeces	invasive genetic sampling	NA	NA	equivalent	[33]
genotyping success	2011	France	mollusc	skin swab	invasive genetic	NA	NA	equivalent	[34]

					sampling				
genotyping success	2018	Austria	amphibian	skin swab	invasive genetic sampling	NA	NA	inferior	[35]
genotyping success	2009	Canada	carnivore	faeces	invasive genetic sampling	NA	NA	equivalent	[36]
genotyping success	2013	UK	arthropod	faeces	invasive genetic sampling	NA	NA	equivalent	[37]
genotyping success	2006	Canada	bird	eggshell	invasive genetic sampling	NA	NA	equivalent	[38]
genotyping success	2020	Canada	reptile	cloacal swab	invasive genetic sampling	NA	NA	equivalent	[39]
genotyping success	2011	Germany	reptile	buccal swab	invasive genetic sampling	NA	lower	equivalent	[40]
genotyping success	2020	Guyana	bird	feather	invasive genetic sampling	NA	NA	equivalent	[41]
genotyping success	2006	USA	carnivore	shed skin	invasive genetic sampling	NA	NA	equivalent	[42]
genotyping success	2017	UK	fish	skin swab	invasive genetic sampling	NA	NA	equivalent	[43]
genotyping success	2011	Japan	bird	eggshell	invasive genetic sampling	NA	NA	equivalent	[44]
species detection	2019	USA	carnivore	hair	camera traps	lower	lower	equivalent	[45]
species detection	2020	Australia	fish	eDNA (water)	field visual or acoustic survey	NA	NA	superior	[46]
species detection	2014	Canada	carnivore	hair	camera traps	NA	NA	inferior	[47]
species detection	2016	Canada	carnivore	hair	camera traps	NA	NA	equivalent	[48]
species detection	2012	Denmark	cetacean	eDNA (water)	field visual or acoustic survey	NA	NA	equivalent	[49]
species detection	2018	Laos	fish	eDNA (water)	lethal sampling	NA	NA	superior	[50]
species detection	2020	USA	arthropod	eDNA (water)	lethal sampling	lower	lower	superior	[51]
species detection	2006	USA	carnivore	faeces	multiple methods	NA	NA	equivalent	[52]
species detection	2019	UK	fish	eDNA (water)	lethal sampling	NA	NA	equivalent	[53]
species detection	2019	Canada	amphibian	eDNA (water)	field visual or acoustic survey	lower	lower	superior	[54]
species detection	2018	France	rodent	hair	live trapping	higher	higher	superior	[55]
species detection	2020	India	multiple species	faeces	multiple methods	NA	NA	equivalent	[56]
species detection	2013	USA	lagomorph	faeces	invasive genetic	lower	lower	superior	[57]

					sampling				
species detection	2017	Brazil	amphibian	eDNA (water)	field visual or acoustic survey	NA	lower	superior	[58]
species detection	2020	Greece	fish	eDNA (water)	lethal sampling	NA	NA	superior	[59]
species detection	2014	Portugal	carnivore	hair	camera traps	NA	NA	inferior	[60]
species detection	2016	USA	amphibian	eDNA (water)	live trapping	lower	NA	superior	[61]
species detection	2020	France	mollusc	eDNA (water)	field visual or acoustic survey	NA	lower	superior	[62]
species detection	2008	Spain	carnivore	faeces	camera traps	NA	lower	superior	[63]
species detection	2020	UK	multiple species	eDNA (water)	multiple methods	NA	lower	equivalent	[64]
species detection	2016	Australia	fish	eDNA (water)	lethal sampling	NA	NA	equivalent	[65]
species detection	2016	Australia	amphibian	eDNA (water)	live trapping	equivalent	lower	equivalent	[66]
species detection	2019	Australia	fish	eDNA (water)	field visual or acoustic survey	NA	NA	equivalent	[67]
species detection	2019	USA	fish	eDNA (water)	field visual or acoustic survey	NA	lower	superior	[68]
species detection	2019	China	cetacean	eDNA (water)	field visual or acoustic survey	NA	NA	superior	[69]
species detection	2015	Italy	carnivore	faeces	camera traps	NA	NA	equivalent	[70]
species detection	2014	Australia	marsupial	faeces	field visual or acoustic survey	lower	lower	superior	[71]
species detection	2020	Ireland	fish	eDNA (water)	lethal sampling	NA	lower	superior	[72]
species detection	2020	China	fish	eDNA (water)	lethal sampling	NA	NA	superior	[73]
population size estimation	2019	France	bird	faeces	field visual or acoustic survey	equivalent	lower	superior	[74]
population size estimation	2007	Sweden	carnivore	faeces	field visual or acoustic survey	higher	NA	superior	[75]
population size estimation	2016	Italy	carnivore	faeces	camera traps	higher	NA	superior	[76]
population size estimation	2018	Canada	carnivore	hair	camera traps	lower	lower	superior	[77]
population size estimation	2011	USA	carnivore	hair	lethal sampling	NA	NA	superior	[78]
population size estimation	2018	Portugal	rodent	faeces	invasive genetic sampling	lower	lower	superior	[79]
population size estimation	2016	Norway	carnivore	faeces	field visual or acoustic survey	NA	NA	superior	[80]

population size estimation	2009	Czech Republic	carnivore	faeces	field visual or acoustic survey	higher	higher	superior	[81]
population size estimation	2013	Laos	elephant	faeces	field visual or acoustic survey	lower	lower	superior	[82]
population size estimation	2017	USA	lagomorph	faeces	live trapping	lower	lower	equivalent	[83]
population size estimation	2011	Mongolia	carnivore	faeces	camera traps	lower	lower	superior	[84]
population size estimation	2013	Canada	carnivore	hair	radiotelemetry	lower	lower	superior	[85]
population size estimation	2018	Iran	carnivore	faeces	questionnaire survey	NA	NA	superior	[86]
population size estimation	2014	Panama	carnivore	faeces	camera traps	NA	NA	equivalent	[87]
population size estimation	2009	Australia	marsupial	faeces	live trapping	NA	higher	equivalent	[88]
population size estimation	2010	Australia	marsupial	hair	live trapping	NA	lower	inferior	[89]
population size estimation	2018	Portugal	rodent	faeces	live trapping	NA	lower	superior	[90]
population size estimation	2019	USA	fish	eDNA (water)	lethal sampling	NA	NA	equivalent	[91]
population size estimation	2006	Sweden	carnivore	faeces	field visual or acoustic survey	lower	NA	superior	[92]
population size estimation	2013	Brazil	carnivore	faeces	camera traps	NA	NA	equivalent	[93]
population size estimation	2011	USA	carnivore	faeces	radiotelemetry	NA	lower	equivalent	[94]
population size estimation	2019	USA	carnivore	hair	multiple methods	NA	NA	superior	[95]
population size estimation	2018	France	bat	faeces	field visual or acoustic survey	NA	NA	superior	[96]
diet analysis	2007	Australia	carnivore	faeces	microhistological analysis	NA	lower	superior	[97]
diet analysis	2007	Australia	bird	faeces	stomach flushing	NA	NA	inferior	[98]
diet analysis	2019	Spain	ungulate	faeces	microhistological analysis	lower	lower	equivalent	[99]
diet analysis	2017	USA	carnivore	faeces	microhistological	NA	NA	superior	[100]

					analysis				
diet analysis	2019	USA	ungulate	faeces	microhistological analysis	NA	lower	superior	[101]
diet analysis	2017	Germany	bird	faeces	microhistological analysis	NA	NA	superior	[102]
species identification	2017	Brazil	ungulate	faeces	faecal morphometry	NA	NA	superior	[103]
species identification	2008	India	carnivore	faeces	faecal morphometry	NA	NA	superior	[104]
species identification	2016	USA	carnivore	faeces	faecal morphometry	NA	NA	superior	[105]
species identification	2019	Brazil	carnivore	faeces	faecal morphometry	NA	NA	superior	[106]
species identification	2008	Gabon	ungulate	faeces	faecal morphometry	NA	NA	superior	[107]
health monitoring	2020	USA	bird	feather	invasive genetic sampling	NA	NA	equivalent	[108]
health monitoring	2015	USA	bird	faeces	microhistological analysis	NA	NA	superior	[109]
health monitoring	2015	USA	bird	faeces	invasive genetic sampling	NA	NA	inferior	[110]
health monitoring	2016	USA	cetacean	blowhole swab	invasive genetic sampling	NA	NA	equivalent	[111]
individual identification	2015	USA	lagomorph	faeces	radiotelemetry	lower	NA	superior	[112]
individual identification	2014	Portugal	fish	skin swab	colouration patterns recognition	higher	higher	equivalent	[113]

References:

1. Bosnjak, J.; Stevanov-Pavlovic, M.; Vucicevic, M.; Stevanovic, J.; Simeunovic, P.; Resanovic, R.; Stanimirovic, Z. Feasibility of non-invasive molecular method for sexing of parrots. *Pak. J. Zool.* **2013**, *45*, 715-720.
2. Boston, E.S.M.; Puechmaille, S.J.; Scott, D.D.; Buckley, D.J.; Lundy, M.G.; Montgomery, I.W.; Prodöhl, P.A.; Teeling, E.C. Empirical assessment of non-invasive population genetics in bats: comparison of DNA quality from faecal and tissue samples. *Acta Chiropt* **2012**, *14*, 45-52.
3. Dai, Y.; Lin, Q.; Fang, W.; Zhou, X.; Chen, X. Noninvasive and nondestructive sampling for avian microsatellite genotyping: a case study on the vulnerable Chinese egret (*Egretta eulophotes*). *Avian Res* **2015**, *6*, 24.

4. De Barba, M.; Adams, J.R.; Goldberg, C.S.; Stansbury, C.R.; Arias, D.; Cisneros, R.; Waits, L.P. Molecular species identification for multiple carnivores. *Conserv. Genet. Resour.* **2014**, *6*, 821-824.
5. Duenas, J.F.; Cruickshank, R.; Ross, J. Optimisation of a microsatellite panel for the individual identification of brushtail possums using low template DNA. *N. Z. J. Ecol.* **2015**, *39*, 93-102.
6. Egloff, C.; Labrosse, A.; Hebert, C.; Crump, D. A nondestructive method for obtaining maternal DNA from avian eggshells and its application to embryonic viability determination in herring gulls (*Larus argentatus*). *Molecular Ecology Resources* **2009**, *9*, 19-27.
7. Eriksson, C.E.; Ruprecht, J.; Levi, T. More affordable and effective noninvasive single nucleotide polymorphism genotyping using high-throughput amplicon sequencing. *Molecular Ecology Resources* **2020**, *20*, 1505-1516.
8. Farro, A.P.C.; Rollo Jr, M.M.; Silva Jr, J.M.; Marino, C.L. A simple protocol for a low invasive DNA accessing in *Stenella longirostris* (Cetacea: Delphinidae). *Panam J Aquatic Sci* **2008**, *3*, 130-134.
9. Fernando, P.; Vidya, T.N.C.; Rajapakse, C.; Dangolla, A.; Melnick, D.J. Reliable noninvasive genotyping: fantasy or reality? *J. Hered.* **2003**, *94*, 115-123.
10. Ford, B.; Govindarajulu, P.; Larsen, K.; Russello, M. Evaluating the efficacy of non-invasive genetic sampling of the Northern Pacific rattlesnake with implications for other venomous squamates. *Conserv. Genet. Resour.* **2017**, *9*, 13-15.
11. Franco-Gutiérrez, L.J.; Álvarez-Cardona, J.; Soto-Calderón, I.D. Sex identification of neotropical macaws (*Ara* spp.) from invasive and non-invasive samples. *Ornitol Colomb* **2017**, *2017*, eNB03.
12. Frosch, C.; Haase, P.; Nowak, C. First set of microsatellite markers for genetic characterization of the Eurasian beaver (*Castor fiber*) based on tissue and hair samples. *Eur J Wildl Res* **2011**, *57*, 679-682.
13. Gallardo, C.E.; Correa, C.; Morales, P.; Saez, P.A.; Pastenes, L.; Mendez, M.A. Validation of a cheap and simple nondestructive method for obtaining AFLPs and DNA sequences (mitochondrial and nuclear) in amphibians. *Molecular Ecology Resources* **2012**, *12*, 1090-1096.
14. Handel, C.M.; Pajot, L.M.; Talbot, S.L.; Sage, G.K. Use of buccal swabs for sampling DNA from nestling and adult birds. *Wildl. Soc. Bull.* **2006**, *34*, 1094-1100.
15. Hedmark, E.; Flagstad, O.; Segerstrom, P.; Persson, J.; Landa, A.; Ellegren, H. DNA-based individual and sex identification from wolverine (*Gulo gulo*) faeces and urine. *Conserv. Genet.* **2004**, *5*, 405-410.
16. Hollenbeck, N.; Scheel, D.; Gravley, M.C.; Sage, G.K.; Toussaint, R.; Talbot, S.L. Use of swabs for sampling epithelial cells for molecular genetics analyses in *Enteroctopus*. *Am. Malacol. Bull.* **2017**, *35*, 145-157.
17. Holman, L.E.; Hollenbeck, C.M.; Ashton, T.J.; Johnston, I.A. Demonstration of the use of environmental DNA for the non-invasive genotyping of a bivalve mollusk, the European flat oyster (*Ostrea edulis*). *Front Genet* **2019**, *10*, 1159.
18. Huang, X.; Zhou, X.; Lin, Q.; Fang, W.; Chen, X. An efficient molecular sexing of the vulnerable Chinese egret (*Egretta eulophotes*) from faeces samples. *Conserv. Genet. Resour.* **2012**, *4*, 391-393.
19. Karlsson, S.; Larsen, B.M.; Eriksen, L.; Hagen, M. Four methods of nondestructive DNA sampling from freshwater pearl mussels *Margaritifera margaritifera* L. (Bivalvia: Unionoida). *Freshw Sci* **2013**, *32*, 525-530.
20. Khan, A.; Patel, K.; Bhattacharjee, S.; Sharma, S.; Chugani, A.N.; Sivaraman, K.; Hosawad, V.; Sahu, Y.K.; Reddy, G.V.; Ramakrishnan, U. Are shed hair genomes the most effective noninvasive resource for estimating relationships in the wild? *Ecology and Evolution* **2020**, *10*, 4583-4594.

21. Lathuilliere, M.; Menard, N.; Gautier-Hion, A.; Crouau-Roy, B. Testing the reliability of noninvasive genetic sampling by comparing analyses of blood and fecal samples in Barbary macaques (*Macaca sylvanus*). *Am. J. Primatol.* **2001**, *55*, 151-158.
22. Maia, T.A.; Vilaca, S.T.; da Silva, L.R.; Santos, F.R.; Dantas, G.P.D. DNA sampling from eggshells and microsatellite genotyping in rare tropical birds: case study on Brazilian merganser. *Genet. Mol. Biol.* **2017**, *40*, 808-812.
23. Martin-Galvez, D.; Peralta-Sanchez, J.M.; Dawson, D.A.; Martin-Platero, A.M.; Martinez-Bueno, M.; Burke, T.; Soler, J.J. DNA sampling from eggshell swabbing is widely applicable in wild bird populations as demonstrated in 23 species. *Molecular Ecology Resources* **2011**, *11*, 481-493.
24. Mitelberg, A.; Vandergast, A.G.; Nussear, K.E.; Dutcher, K.; Esque, T.C. Development of a genotyping protocol for Mojave Desert tortoise scat. *Chelonian Conserv. Biol.* **2019**, *18*, 123-132.
25. Nussberger, B.; Wandeler, P.; Camenisch, G. A SNP chip to detect introgression in wildcats allows accurate genotyping of single hairs. *Eur J Wildl Res* **2014**, *60*, 405-410.
26. Ogden, R.; Langenhorst, T.; McEwing, R.; Woodfine, T. Genetic markers and sample types for pedigree reconstruction in Grevy's zebra (*Equus grevyi*). *Der Zoologische Garten* **2007**, *77*, 29-35.
27. Olah, G.; Heinsohn, R.G.; Brightsmith, D.J.; Espinoza, J.R.; Peakall, R. Validation of non-invasive genetic tagging in two large macaw species (*Ara macao* and *A. chloropterus*) of the Peruvian Amazon. *Conserv. Genet. Resour.* **2016**, *8*, 499-509.
28. Ozana, S.; Pyszko, P.; Dolny, A. Determination of suitable insect part for non-lethal DNA sampling: case study of DNA quality and regeneration capability of dragonflies. *Insect Conserv Diver* **2020**, *13*, 319-327.
29. Pearce, J.M.; Fields, R.L.; Scribner, K.T. Nest materials as a source of genetic data for avian ecological studies. *J. Field Ornithol.* **1997**, *68*, 471-481.
30. Peralta, D.M.; Ibanez, E.A.; Lucero, S.; Cappozzo, H.L.; Tunez, J.I. A new minimally invasive and inexpensive sampling method for genetic studies in pinnipeds. *Mamm. Res.* **2020**, *65*, 11-18.
31. Perry, G.H.; Marioni, J.C.; Melsted, P.; Gilad, Y. Genomic-scale capture and sequencing of endogenous DNA from feces. *Mol. Ecol.* **2010**, *19*, 5332-5344.
32. Pichlmuller, F.; Straub, C.; Helfer, V. Skin swabbing of amphibian larvae yields sufficient DNA for efficient sequencing and reliable microsatellite genotyping. *Amphibia-Reptilia* **2013**, *34*, 517-523.
33. Powell, C.; Butler, F.; O'Reilly, C. The development of real-time PCR assays for species and sex identification of three sympatric deer species from noninvasive samples. *Conserv. Genet. Resour.* **2019**, *11*, 465-471.
34. Régnier, C.; Gargominy, O.; Falkner, G.; Puillandre, N. Foot mucus stored on FTA® cards is a reliable and non-invasive source of DNA for genetics studies in molluscs. *Conserv. Genet. Resour.* **2011**, *3*, 377-382.
35. Ringler, E. Testing skin swabbing for DNA sampling in dendrobatid frogs. *Amphibia-Reptilia* **2018**, *39*, 245-251.
36. Rutledge, L.Y.; Holloway, J.J.; Patterson, B.R.; White, B.N. An improved field method to obtain DNA for individual identification from wolf scat. *J. Wildl. Manage.* **2009**, *73*, 1430-1435.
37. Scriven, J.J.; Woodall, L.C.; Goulson, D. Nondestructive DNA sampling from bumblebee faeces. *Molecular Ecology Resources* **2013**, *13*, 225-229.
38. Schmaltz, G.; Somers, C.M.; Sharma, P.; Quinn, J.S. Non-destructive sampling of maternal DNA from the external shell of bird eggs. *Conserv. Genet.* **2006**, *7*, 543-549.
39. Schmidt, D.A.; Campbell, N.R.; Govindarajulu, P.; Larsen, K.W.; Russello, M.A. Genotyping-in-Thousands by sequencing (GT-seq) panel development and application to minimally invasive DNA samples to support studies in molecular ecology. *Molecular Ecology Resources* **2020**, *20*, 114-124.

40. Schulte, U.; Gebhard, F.; Heinz, L.; Veith, M.; Hochkirch, A. Buccal swabs as a reliable non-invasive tissue sampling method for DNA analysis in the lacertid lizard *Podarcis muralis*. *North-West J Zool* **2011**, *7*, 325-328.
41. Spitzer, R.; Norman, A.J.; Konigsson, H.; Schiffthaler, B.; Spong, G. De novo discovery of SNPs for genotyping endangered sun parakeets (*Aratinga solstitialis*) in Guyana. *Conserv. Genet. Resour.* **2020**, *12*, 631-641.
42. Swanson, B.J.; Kelly, B.P.; Maddox, C.K.; Moran, J.R. Shed skin as a source of DNA for genotyping seals. *Mol. Ecol. Notes* **2006**, *6*, 1006-1009.
43. Taslima, K.; Taggart, J.B.; Wehner, S.; McAndrew, B.J.; Penman, D.J. Suitability of DNA sampled from Nile tilapia skin mucus swabs as a template for ddRAD-based studies. *Conserv. Genet. Resour.* **2017**, *9*, 39-42.
44. Urano, K.; Yamada, T.; Taniguchi, Y.; Iwaisaki, H.; Sugiyama, T.; Homma, K.; Kaneko, Y.; Yamagishi, S. Non-invasive sampling technique for DNA extraction from captive Japanese crested ibis on Sado Island. *Anim Sci J* **2011**, *82*, 616-619.
45. Alldredge, M.W.; Blecha, T.; Lewis, J.H. Less invasive monitoring of cougars in colorado's front range. *Wildl. Soc. Bull.* **2019**, *43*, 222-230.
46. Bessey, C.; Jarman, S.N.; Berry, O.; Olsen, Y.S.; Bunce, M.; Simpson, T.; Power, M.; McLaughlin, J.; Edgar, G.J.; Keesing, J. Maximizing fish detection with eDNA metabarcoding. *Environ DNA* **2020**, *2*, 493-504.
47. Fisher, J.T.; Bradbury, S. A multi-method hierarchical modeling approach to quantifying bias in occupancy from noninvasive genetic tagging studies. *J. Wildl. Manage.* **2014**, *78*, 1087-1095.
48. Fisher, J.T.; Heim, N.; Code, S.; Paczkowski, J. Grizzly bear noninvasive genetic tagging surveys: estimating the magnitude of missed detections. *PLoS ONE* **2016**, *11*, e0161055.
49. Foote, A.D.; Thomsen, P.F.; Sveegaard, S.; Wahlberg, M.; Kielgast, J.; Kyhn, L.A.; Salling, A.B.; Galatius, A.; Orlando, L.; Gilbert, M.T.P. Investigating the potential use of environmental DNA (eDNA) for genetic monitoring of marine mammals. *PLoS ONE* **2012**, *7*, e41781.
50. Gillet, B.; Cottet, M.; Destanque, T.; Kue, K.; Descloux, S.; Chanudet, V.; Hughes, S. Direct fishing and eDNA metabarcoding for biomonitoring during a 3-year survey significantly improves number of fish detected around a South East Asian reservoir. *PLoS ONE* **2018**, *13*, e0208592.
51. Gold, Z.; Wall, A.R.; Curd, E.E.; Kelly, R.P.; Pentcheff, N.D.; Ripma, L.; Barber, P.H.; Wetzer, R. eDNA metabarcoding bioassessment of endangered fairy shrimp (*Branchinecta* spp.). *Conserv. Genet. Resour.* **2020**, *12*, 685-690.
52. Gompper, M.E.; Kays, R.W.; Ray, J.C.; Lapoint, S.D.; Bogan, D.A.; Cryan, J.R. A comparison of noninvasive techniques to survey carnivore communities in northeastern North America. *Wildl. Soc. Bull.* **2006**, *34*, 1142-1151.
53. Harper, L.R.; Bernd, H.n.; Sayer, C.D.; Read, D.S.; Li, J.; Harper, K.J.; Lori Lawson, H.; Griffiths, N.P.; Blackman, R.C. Development and application of environmental DNA surveillance for the threatened crucian carp (*Carassius carassius*). *Freshwat. Biol.* **2019**, *64*, 93-107.
54. Hobbs, J.; Round, J.M.; Allison, M.J.; Helbing, C.C. Expansion of the known distribution of the coastal tailed frog, *Ascaphus truei*, in British Columbia, Canada, using robust eDNA detection methods. *PLoS ONE* **2019**, *14*, e0213849.
55. Chiron, F.; Hein, S.; Charge, R.; Julliard, R.; Martin, L.; Roguet, A.; Jacob, J. Validation of hair tubes for small mammal population studies. *J. Mammal.* **2018**, *99*, 478-485.
56. Joshi, B.D.; Sharief, A.; Kumar, V.; Kumar, M.; Dutta, R.; Devi, R.; Singh, A.; Thakur, M.; Sharma, L.K.; Chandra, K. Field testing of different methods for monitoring mammals in Trans-Himalayas: a case study from Lahaul and Spiti. *Glob Ecol Conserv* **2020**, *21*, e00824.

57. Kilpatrick, H.J.; Goodie, T.J.; Kovach, A.I. Comparison of live-trapping and noninvasive genetic sampling to assess patch occupancy by New England cottontail (*Sylvilagus transitionalis)* rabbits. *Wildl. Soc. Bull.* **2013**, *37*, 901-905.
58. Lopes, C.M.; Sasso, T.; Valentini, A.; Dejean, T.; Martins, M.; Zamudio, K.R.; Haddad, C.F.B. eDNA metabarcoding: a promising method for anuran surveys in highly diverse tropical forests. *Molecular Ecology Resources* **2017**, *17*, 904-914.
59. Mauvisseau, Q.; Kalogianni, E.; Zimmerman, B.; Bulling, M.; Brys, R.; Sweet, M. eDNA-based monitoring: advancement in management and conservation of critically endangered killifish species. *Environ DNA* **2020**, *2*, 601-613.
60. Monterroso, P.; Rich, L.N.; Serronha, A.; Ferreras, P.; Alves, P.C. Efficiency of hair snares and camera traps to survey mesocarnivore populations. *Eur J Wildl Res* **2014**, *60*, 279-289.
61. Pierson, T.W.; McKee, A.M.; Spear, S.F.; Maerz, J.C.; Camp, C.D.; Glenn, T.C. Detection of an enigmatic plethodontid salamander using environmental DNA. *Copeia* **2016**, *104*, 78-82.
62. Prié, V.; Valentini, A.; Lopes-Lima, M.; Froufe, E.; Rocle, M.; Poulet, N.; Taberlet, P.; Dejean, T. Environmental DNA metabarcoding for freshwater bivalves biodiversity assessment: methods and results for the Western Palearctic (European sub-region). *Hydrobiologia* **2020**, *848*, 2931-2950.
63. Rosellini, S.; Osorio, E.; Ruiz-Gonzalez, A.; Isabel, A.P.; Barja, I. Monitoring the small-scale distribution of sympatric European pine martens (*Martes martes*) and stone martens (*Martes foina*): a multievidence approach using faecal DNA analysis and camera-traps. *Wildl. Res.* **2008**, *35*, 434-440.
64. Sales, N.G.; McKenzie, M.B.; Drake, J.; Harper, L.R.; Browett, S.S.; Coscia, I.; Wangensteen, O.S.; Baillie, C.; Bryce, E.; Dawson, D.A., et al. Fishing for mammals: Landscape-level monitoring of terrestrial and semi-aquatic communities using eDNA from riverine systems. *J. Appl. Ecol.* **2020**, *57*, 707-716.
65. Shaw, J.L.A.; Clarke, L.J.; Wedderburn, S.D.; Barnes, T.C.; Weyrich, L.S.; Cooper, A. Comparison of environmental DNA metabarcoding and conventional fish survey methods in a river system. *Biol. Conserv.* **2016**, *197*, 131-138.
66. Smart, A.S.; Weeks, A.R.; van Rooyen, A.R.; Moore, A.; McCarthy, M.A.; Tingley, R. Assessing the cost-efficiency of environmental DNA sampling. *Methods in Ecology and Evolution* **2016**, *7*, 1291-1298.
67. Stat, M.; Harvey, E.S.; Jeffrey, J.; DiBattista, J.D.; Bunce, M.; Newman, S.J. Combined use of eDNA metabarcoding and video surveillance for the assessment of fish biodiversity. *Conserv. Biol.* **2019**, *33*, 196-205.
68. Strickland, G.J.; Roberts, J.H. Utility of eDNA and occupancy models for monitoring an endangered fish across diverse riverine habitats. *Hydrobiologia* **2019**, *826*, 129-144.
69. Tang, Y.; Wu, Y.; Liu, K.; Li, J.; Li, H.; Wang, Q.; Yu, J.; Pao, X. Investigating the distribution of the Yangtze finless porpoise in the Yangtze River using environmental DNA. *PLoS ONE* **2019**, *14*, e0221120.
70. Velli, E.; Bologna, M.A.; Silvia, C.; Ragni, B.; Randi, E. Non-invasive monitoring of the European wildcat (*Felis silvestris silvestris* Schreber, 1777): comparative analysis of three different monitoring techniques and evaluation of their integration. *Eur J Wildl Res* **2015**, *61*, 657-668.
71. Wadley, J.J.; Austin, J.J.; Fordham, D.A. Genetic inference as a method for modelling occurrence: a viable alternative to visual surveys. *Austral Ecol.* **2014**, *39*, 952-962.
72. Weldon, L.; Ciara, O.L.; Steer, M.; Newton, L.; Macdonald, H.; Sargeant, S.L. A comparison of European eel *Anguilla anguilla* eDNA concentrations to fyke net catches in five Irish lakes. *Environ DNA* **2020**, *2*, 587-600.
73. Zou, K.S.; Chen, J.W.; Ruan, H.T.; Li, Z.H.; Guo, W.J.; Li, M.; Liu, L. eDNA metabarcoding as a promising conservation tool for monitoring fish diversity in a coastal wetland of the Pearl River Estuary compared to bottom trawling. *Sci. Total Environ.* **2020**, *702*, 134704.

74. Aleix-Mata, G.; Adrados, B.; Boos, M.; Marty, E.; Mourieres, P.; Tucat, G.; Thion, N.; Mossoll-Torres, M.; Pérez, J.M.; Sánchez, A. Comparing methods for estimating the abundance of western capercaillie *Tetrao urogallus* males in Pyrenean leks: singing counts versus genetic analysis of non-invasive samples. *Bird Study* **2019**, *66*, 565-569.
75. Arrendal, J.; Vila, C.; Bjorklund, M. Reliability of noninvasive genetic census of otters compared to field censuses. *Conserv. Genet.* **2007**, *8*, 1097-1107.
76. Balestrieri, A.; Ruiz-González, A.; Vergara, M.; Capelli, E.; Tirozzi, P.; Alfino, S.; Minuti, G.; Prigioni, C.; Saino, N. Pine marten density in lowland riparian woods: a test of the Random Encounter Model based on genetic data. *Mamm. Biol.* **2016**, *81*, 439-446.
77. Burgar, J.M.; Stewart, F.E.C.; Volpe, J.P.; Fisher, J.T.; Burton, A.C. Estimating density for species conservation: comparing camera trap spatial count models to genetic spatial capture-recapture models. *Glob Ecol Conserv* **2018**, *15*.
78. Coster, S.S.; Kovach, A.I.; Pekins, P.J.; Cooper, A.B.; Timmins, A. Genetic mark-recapture population estimation in black bears and issues of scale. *J. Wildl. Manage.* **2011**, *75*, 1128-1136.
79. Ferreira, C.M.; Sabino-Marques, H.; Barbosa, S.; Costa, P.; Encarnação, C.; Alpizar-Jara, R.; Pita, R.; Beja, P.; Mira, A.; Searle, J.B., et al. Genetic non-invasive sampling (gNIS) as a cost-effective tool for monitoring elusive small mammals. *Eur J Wildl Res* **2018**, *64*, 46.
80. Gervasi, V.; Broseth, H.; Gimenez, O.; Nilsen, E.B.; Odden, J.; Flagstad, O.; Linnell, J.D.C. Sharing data improves monitoring of trans-boundary populations: the case of wolverines in central Scandinavia. *Wildl. Biol.* **2016**, *22*, 95-106.
81. Hajkova, P.; Zemanova, B.; Roche, K.; Hajek, B. An evaluation of field and noninvasive genetic methods for estimating Eurasian otter population size. *Conserv. Genet.* **2009**, *10*, 1667-1681.
82. Hedges, S.; Johnson, A.; Ahlering, M.; Tyson, M.; Eggert, L.S. Accuracy, precision, and cost-effectiveness of conventional dung density and fecal DNA based survey methods to estimate Asian elephant (*Elephas maximus*) population size and structure. *Biol. Conserv.* **2013**, *159*, 101-108.
83. Cheng, E.; Hodges, K.E.; Sollmann, R.; Mills, L.S. Genetic sampling for estimating density of common species. *Ecology and Evolution* **2017**, *7*, 6210-6219.
84. Janecka, J.E.; Munkhtsog, B.; Jackson, R.M.; Naranbaatar, G.; Mallon, D.P.; Murphy, W.J. Comparison of noninvasive genetic and camera-trapping techniques for surveying snow leopards. *J. Mammal.* **2011**, *92*, 771-783.
85. Johnson, C.J.; Hodder, D.P.; Crowley, S. Assessing noninvasive hair and fecal sampling for monitoring the distribution and abundance of river otter. *Ecol. Res.* **2013**, *28*, 881-892.
86. Moqanaki, E.M.; Jiménez, J.; Bensch, S.; José Vicente, L.-B. Counting bears in the Iranian Caucasus: remarkable mismatch between scientifically-sound population estimates and perceptions. *Biol. Conserv.* **2018**, *220*, 182-191.
87. Rodgers, T.W.; Giacalone, J.; Heske, E.J.; Janecka, J.E.; Phillips, C.A.; Schooley, R.L. Comparison of noninvasive genetics and camera trapping for estimating population density of ocelots (*Leopardus pardalis*) on Barro Colorado Island, Panama. *Trop Conserv Sci* **2014**, *7*, 690-705.
88. Ruibal, M.; Peakall, R.; Claridge, A.; Firestone, K. Field-based evaluation of scat DNA methods to estimate population abundance of the spotted-tailed quoll (*Dasyurus maculatus*), a rare Australian marsupial. *Wildl. Res.* **2009**, *36*, 721-736.
89. Ruibal, M.; Peakall, R.; Claridge, A.; Murray, A.; Firestone, K. Advancement to hair-sampling surveys of a medium-sized mammal: DNA-based individual identification and population estimation of a rare Australian marsupial, the spotted-tailed quoll (*Dasyurus maculatus*). *Wildl. Res.* **2010**, *37*, 27-38.
90. Sabino-Marques, H.; Clara Mendes, F.; Paupério, J.; Costa, P.; Barbosa, S.; Encarnação, C.; Alpizar-Jara, R.; Alves, P.C.; Searle, J.B.; Mira, A., et al. Combining genetic non-invasive sampling with spatially explicit capture-recapture models for density estimation of a patchily distributed small mammal. *Eur J Wildl Res* **2018**, *64*, 44.

91. Shelton, A.O.; Kelly, R.P.; O'Donnell, J.L.; Park, L.; Schwenke, P.; Greene, C.; Henderson, R.A.; Beamer, E.M. Environmental DNA provides quantitative estimates of a threatened salmon species. *Biol. Conserv.* **2019**, *237*, 383-391.
92. Solberg, K.H.; Bellemain, E.; Drageset, O.M.; Taberlet, P.; Swenson, J.E. An evaluation of field and non-invasive genetic methods to estimate brown bear (*Ursus arctos*) population size. *Biol. Conserv.* **2006**, *128*, 158-168.
93. Sollmann, R.; Torres, N.M.; Furtado, M.M.; de Almeida Jacomo, A.T.; Palomares, F.; Roques, S.; Silveira, L. Combining camera-trapping and noninvasive genetic data in a spatial capture-recapture framework improves density estimates for the jaguar. *Biol. Conserv.* **2013**, *167*, 242-247.
94. Stenglein, J.L.; Waits, L.P.; Ausband, D.E.; Zager, P.; Mack, C.M. Estimating gray wolf pack size and family relationships using noninvasive genetic sampling at rendezvous sites. *J. Mammal.* **2011**, *92*, 784-795.
95. Welfelt, L.S.; Beausoleil, R.A.; Wielgus, R.B. Factors associated with black bear density and implications for management. *J. Wildl. Manage.* **2019**, *83*, 1527-1539.
96. Zarzoso-Lacoste, D.; Pierre-Loup, J.; Lehnen, L.; Girard, T.; Anne-Laure, B.; Puechmaille, S.J.; Petit, E.J. Combining noninvasive genetics and a new mammalian sex-linked marker provides new tools to investigate population size, structure and individual behaviour: an application to bats. *Molecular Ecology Resources* **2018**, *18*, 217-228.
97. Casper, R.M.; Jarrnan, S.N.; Deagle, B.E.; Gales, N.J.; Hindell, M.A. Detecting prey from DNA in predator scats: a comparison with morphological analysis, using *Arctocephalus* seals fed a known diet. *J. Exp. Mar. Biol. Ecol.* **2007**, *347*, 144-154.
98. Deagle, B.E.; Gales, N.J.; Evans, K.; Jarman, S.N.; Robinson, S.; Trebilco, R.; Hindell, M.A. Studying seabird diet through genetic analysis of faeces: a case study on macaroni penguins (*Eudyptes chrysolophus*). *PLoS ONE* **2007**, *2*, e831.
99. Espunyes, J.; Espunya, C.; Chaves, S.; Calleja, J.A.; Bartolomé, J.; Serrano, E. Comparing the accuracy of PCR-capillary electrophoresis and cuticle microhistological analysis for assessing diet composition in ungulates: a case study with Pyrenean chamois. *PLoS ONE* **2019**, *14*, e0216345.
100. Gosselin, E.N.; Lonsinger, R.C.; Waits, L.P. Comparing morphological and molecular diet analyses and fecal DNA sampling protocols for a terrestrial carnivore. *Wildl. Soc. Bull.* **2017**, *41*, 362-369.
101. King, S.R.B.; Schoenecker, K.A. Comparison of methods to examine diet of feral horses from noninvasively collected fecal samples. *Rangeland Ecol. Manage.* **2019**, *72*, 661-666.
102. Thalinger, B.; Oehm, J.; Obwexer, A.; Traugott, M. The influence of meal size on prey DNA detectability in piscivorous birds. *Molecular Ecology Resources* **2017**, *17*, e174-e186.
103. Costa, E.B.V.; de Oliveira, M.L.; Peres, P.H.D.; Grotta-Neto, F.; Vogliotti, A.; Piovezan, U.; Duarte, J.M.B. Low accuracy of identifying Neotropical deer species by scat morphology. *Stud Neotrop Fauna Environ* **2017**, *52*, 37-42.
104. Janecka, J.E.; Jackson, R.; Yuquang, Z.; Diqiang, L.; Munkhtsog, B.; Buckley-Beason, V.; Murphy, W.J. Population monitoring of snow leopards using noninvasive collection of scat samples: a pilot study. *Anim. Conserv.* **2008**, *11*, 401-411.
105. Morin, D.J.; Higdon, S.D.; Holub, J.L.; Montague, D.M.; Fies, M.L.; Waits, L.P.; Kelly, M.J. Bias in carnivore diet analysis resulting from misclassification of predator scats based on field identification. *Wildl. Soc. Bull.* **2016**, *40*, 669-677.
106. Tirelli, F.P.; de Freitas, T.R.O.; Michalski, F.; Percequillo, A.R.; Eizirik, E. Using reliable predator identification to investigate feeding habits of Neotropical carnivores (Mammalia, Carnivora) in a deforestation frontier of the Brazilian Amazon. *Mammalia* **2019**, *83*, 415-427.

107. van Vliet, N.; Zundel, S.; Miquel, C.; Taberlet, P.; Nasi, R. Distinguishing dung from blue, red and yellow-backed duikers through noninvasive genetic techniques. *Afr. J. Ecol.* **2008**, *46*, 411-417.
108. Baek, H.E.; Bandivadekar, R.R.; Pandit, P.; Mah, M.; Sehgal, R.N.M.; Tell, L.A. TaqMan quantitative real-time PCR for detecting avipoxvirus DNA in various sample types from hummingbirds. *PLoS ONE* **2020**, *15*, e0230701.
109. Bertram, M.R.; Hamer, G.L.; Snowden, K.F.; Hartup, B.K.; Hamer, S.A. Coccidian parasites and conservation implications for the endangered whooping crane (*Grus americana*). *PLoS ONE* **2015**, *10*, e0127679.
110. Martinsen, E.S.; Brightman, H.; Fleischer, R.C. Fecal samples fail in PCR-based diagnosis of malaria parasite infection in birds. *Conserv. Genet. Resour.* **2015**, *7*, 15-17.
111. Wu, Q.; Conway, J.; Phillips, K.M.; Stolen, M.; Durden, W.N.; Fauquier, D.; McFee, W.E.; Schwacke, L. Detection of *Brucella* spp. in bottlenose dolphins *Tursiops truncatus* by a real-time PCR using blowhole swabs. *Dis. Aquat. Org.* **2016**, *120*, 241-244.
112. DeMay, S.M.; Rachlow, J.L.; Waits, L.P.; Becker, P.A. Comparing telemetry and fecal DNA sampling methods to quantify survival and dispersal of juvenile pygmy rabbits. *Wildl. Soc. Bull.* **2015**, *39*, 413-421.
113. Monteiro, N.M.; Silva, R.M.; Cunha, M.; Antunes, A.; Jones, A.G.; Vieira, M.N. Validating the use of colouration patterns for individual recognition in the worm pipefish using a novel set of microsatellite markers. *Molecular Ecology Resources* **2014**, *14*, 150-156.
